# Point-of-Care Diagnostic Device for Traumatic Pneumothorax: Low Sensitivity of the Unblinded PneumoScan™

**DOI:** 10.1155/2018/7307154

**Published:** 2018-04-01

**Authors:** M. Rehfeldt, A. Slagman, B. A. Leidel, M. Möckel, T. Lindner

**Affiliations:** ^1^Department of Anaesthesia & Intensive Care, Charité-Universitätsmedizin Berlin, Campus Virchow Klinikum, Berlin, Germany; ^2^Department of Emergency Medicine, Charité-Universitätsmedizin Berlin, Campus Virchow Klinikum, Berlin, Germany; ^3^Department of Emergency Medicine, Charité-Universitätsmedizin Berlin, Campus Benjamin Franklin, Berlin, Germany

## Abstract

**Background:**

Traumatic Pneumothorax (PTX) is a potentially life-threatening injury. It requires a fast and accurate diagnosis and treatment, but diagnostic tools are limited. A new point-of-care device (PneumoScan) based on micropower impulse radar (MIR) promises to diagnose a PTX within seconds. In this study, we compare standard diagnostics with PneumoScan during shock-trauma-room management.

**Patients and Methods:**

Patients with blunt or penetrating chest trauma were consecutively included in the study. All patients were examined including clinical examination with auscultation (CE) and supine chest radiography (CXR). In addition, PneumoScan-readings and thoracic ultrasound scan (US) were performed. Computed tomography (CT) served as gold standard.

**Results:**

CT scan revealed PTX in 11 patients. PneumoScan detected two PTX correctly but missed nine. 15 false-positive results were found by PneumoScan, leading to a sensitivity of 20% and specificity of 80%. Six PTX were detected through CE (sensitivity: 54,5%). CXR detected four (sensitivity: 27,3%) and thoracic US two PTX correctly (sensitivity: 25%).

**Conclusion:**

The unblinded PneumoScan prototype did not confirm the promising results of previous studies. The examined standard diagnostics and thoracic US showed rather weak sensitivity as well. Until now, there is no appropriate point-of-care tool to rule out PTX.

## 1. Introduction

According to the annual report of the TraumaRegister® of the German Trauma Society (DGU), 59,2% of 42954 documented severe trauma patients (*Injury Severity Score (ISS)* ≥ 16 points) showed major thoracic injuries (*Abbreviated Injury Scale (AIS)* ≥ 2 points) [1]. With an incidence of 37–59%, tension Pneumothorax (PTX) is one of the most frequent thoracic injuries [2]. Untreated it leads to mediastinal shifting and consecutive vena-cava-compression. Therefore, all traumatic PTX should be diagnosed at an early stage, especially as treatment is possible in almost all settings.

Since point-of-care diagnostic tools are limited, a fast and accurate diagnosis of PTX is ambitious. Emergency physicians or paramedics can only assess chest-stability, crepitation of broken ribs, and possible skin emphysema. Prominent external jugular veins might indicate a tension PTX as well. Reduced breathing sounds are evaluated by auscultation, which shows weak sensitivity and specificity and is highly examiner-dependent [3].

In shock-trauma-room anterior-posterior chest radiography (CXR) in supine position and computed tomography (CT) scan are part of the routine trauma work-up. While CT scan reveals even PTX of just a few milliliters' volume [4, 5], supine CXR has a rather low sensitivity concerning the detection of small PTX [3, 6–8].

But even a low-volume PTX can develop into a life-threatening tension PTX, observed especially in positive-pressure ventilated patients [5]. A reassessment in the course of time therefore is of paramount importance. To minimise the risk of tension PTX and optimise the treatment of severely injured patients, a fast, reliable, and accurate point-of-care device to rule out PTX is desirable.

PneumoSonic Inc. (Cleveland, OH, USA) introduced the micropower impulse radar (MIR) based PneumoScan (CE-certificate 561036) to detect or exclude PTX within seconds. The MIR-transceiver (connected to a handheld computer) rapidly sends out ultrawideband- (UWB-) waves. Tissue reflections are analyzed in real time by the in-built receiver. PneumoSonic Inc. advertise their device as being examiner independent and easy to handle. It could offer a significant improvement of preclinical PTX-detection and exclusion.

Preliminary investigations with a blinded prototype (clinical data were retrospectively extracted and analyzed) found promising sensitivity and specificity (Table 1) [9–12]. In 2015, Hocagil et al. were the first to publish a study with a proper working, unblinded prototype of PneumoScan [13].

The aim of this study was to conduct a case series with an unblinded PneumoScan and to implement it into shock-trauma-room management. Further on, results were compared to results of standard diagnostics (CXR and clinical examination) and to ultrasound. CT scan was used as gold standard.

## 2. Patients and Methods

### 2.1. Study Procedure

Severely injured adult patients with blunt or penetrating chest trauma were consecutively enrolled at Charité-Universitätsmedizin Berlin, Campus Virchow Klinikum, a level one trauma centre between August 2012 and February 2015. Inclusion criteria are shown in Table 2 (ethics commission vote: EA/091/11).

**Figure 1:**
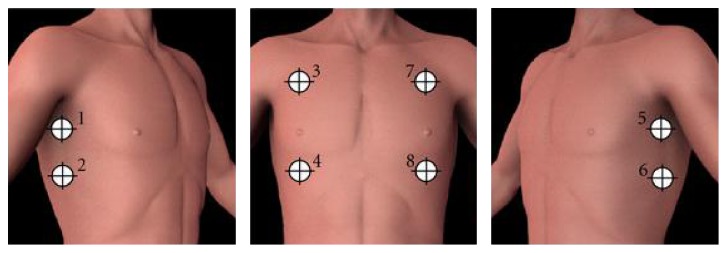
Scan locations of PneumoScan.

Treatment was standardized according to* Advanced Trauma Life Support- (ATLS*®-) protocol. After clinical examination and auscultation, CXR (anterior-posterior) as primary imaging was conducted in shock trauma room. After an initial tutorial of approximately 15 minutes, the study physician was able to perform a PneumoScan reading during primary survey according to the instructions of the device. Therefore, the examiner scanned eight predefined positions (Figure 1).

**Figure 2:**
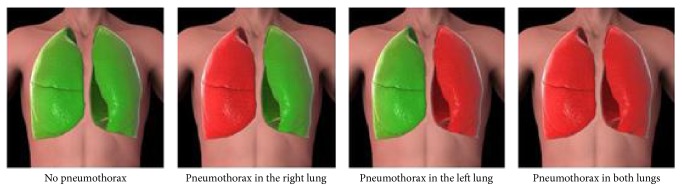
Results displayed by PneumoScan.

PneumoScan readings were immediately displayed to the examiner (unblinded results, Figure 2).

US was performed permanently after six cases, since the first ethical vote did not cover an additional US and therefore an amendment had to be made. Thoracic US readings (Core Vision Pro manufactured in 1998, Toshiba, Minato, Tokyo, Japan) were conducted by two physicians, of which one was trained according to DEGUM (German Society for Ultrasound in Medicine) guidelines.

As recommended, the absence of lung-sliding and comet-tail-artefacts defined PTX [3]. Therefore, a linear B-mode transducer was used, scanning the same eight positions predefined by PneumoScan (Figure 2). Unfortunately, there was no M-mode option on the ultrasound machine.

Results of PneumoScan and thoracic US had no influence on PTX-treatment. After diagnostic work-up and primary treatment the patient underwent whole-body CT scan with contrast agent. Results of CT scan were defined as gold standard to rule out PTX. The clinical significance of PTX was assessed by the trauma team under consideration of the patient's condition and the mechanism of trauma.

Each patient was visited after 18 to 24 hours of admission to the hospital, to check for any adverse events (e.g., erythema) and to obtain the consent to participate in the study. If a patient was not able to consent, for example, due to invasive ventilation, legal proxy was informed.

Once all results of a patient were collected, data was analyzed by the study physicians. All false-positive and false-negative results of PneumoScan-readings were shared with the company to identify possible technical weaknesses. As a consequence, PneumoSonic Inc. decided to change the prototype and to improve the in-built antenna. In total, two device-changes were initialised by PneumoSonic Inc., each with revised software algorithms (Table 6).

To identify possible biases, pulmonary diseases which might interfere with PneumoScan-readings were retrospectively analyzed, as well as the body mass index of each patient and a possible impact of mechanical ventilation.

The statistical analysis was conducted with SPSS version 20.0 (Chicago, IL) for Macintosh. To examine the collected data, nonparametric tests (Mann–Whitney *U* and Chi-Squared) were used. Statistical significance was set to *p* ≤ 0,05.

## 3. Results

### 3.1. Patient Characteristics

In total, 80 injured patients with blunt or penetrating chest trauma were enrolled (24 female, 56 male). Mean age was 47 years. Severity of injury was significantly higher in patients with diagnosed PTX (ISS 29,0 [20,0/41,0]) than in patients without PTX (ISS 12,0 [9,0/22,0]) (*p* = 0,001). Values of vital signs and patient characteristics are shown in Table 3.

11 patients were ventilated at the time of admission, and five got intubated during primary survey. Three of all ventilated patients had a PTX diagnosed.

11 patients presented a PTX, 9 of them after blunt chest trauma, two after penetrating trauma. In three cases, bilateral PTX was diagnosed. All bilateral PTX were analyzed as one case instead of a singular analysis of each hemithorax.

In eight cases PTX was graded as “clinically significant” and therefore treated with chest tube placement. All bilateral PTX were classified as significant by the trauma team.

11 patients presented clinical signs of PTX (CE with auscultation), of which six PTX were confirmed by CT. Three PTX were diagnosed by CXR and only two by ultrasound.

PneumoScan readings were able to be analyzed in 93,8% of all cases (*n* = 75); in 6,3% the device displayed “invalid data” (*n* = 5). PneumoScan managed to detect two PTX but assumed PTX 13 times, which were not confirmed by CT scan (false positive).

Six PTX were missed by standard diagnostics and only detected by CT, thus called occult PTX.

Table 4 gives an overview of the diagnostic accuracy of all examined diagnostic methods.

### 3.2. Potential Disturbing Conditions and Device Replacements

Due to many false-positive and false-negative readings of PneumoScan, potential disturbing conditions of PneumoScan-readings were analyzed. Therefore, all readings were summarized in the groups “correct readings” (all right positive and right negative results, *n* = 54) and “false readings” (all false-positive and false-negative results, *n* = 26). Except mediastinal shifting (*n* = 3; *p* = 0,039) no examined condition had an influence on PneumoScan-readings. Particularly obesity did not significantly affect the results (*p* = 0,7) (Table 5).

After consultation with the developer (PneumoSonic Inc. (Cleveland, OH, USA)), a device replacement was carried out twice. The exchange units were identical but had revised antennas and software algorithms. A single analysis of all three devices used in the study is summarized in Table 6.

### 3.3. Time Schedule

Average time from admission to CT was 23 minutes (20,0/30,0). CE took approximately 2 minutes. Until CXR pictures were ready for interpretation, approximately 8 minutes passed by. To perform a bilateral thoracic ultrasound, the examiner needed about 2 minutes. PneumoScan revealed results within a minute.

### 3.4. Adverse Events

No adverse events (e.g., erythema, pruritus) were documented during routine patient visit 18–24 hours after admission to shock trauma room.

## 4. Discussion

The present study is the first investigation of an unblinded PneumoScan prototype, implemented in a shock-trauma-room algorithm. The present study was not able to confirm the positive findings regarding the sufficient diagnostic value of PneumoScan in previously published data.

Between 2011 and 2013 Levy et al., Albers et al., van der Wilden et al., and Lindner et al. published promising results of PneumoScan, using a blinded device (Table 1). After these positive results, further studies with an unblinded prototype were needed to test the new point-of-care approach under more realistic conditions.

Hocagil et al. were the first to publish a case study with a proper working, unblinded prototype of PneumoScan in 2015 [13]. The authors included 115 patients with suspected PTX, of which only 45 patients were admitted to the hospital due to trauma. Patients with unstable vital signs or tension PTX were excluded. All patients underwent CT scan as gold standard. The authors found a high rate of false-positive readings, leading to a weak specificity. The sensitivity was comparable to previous studies (Table 1).

Comparing the methods of all conducted studies regarding PneumoScan and its diagnostic value, the major difference appears to be the use of an unblinded prototype. While all studies conducted with a blinded unit delivered satisfying results, diagnostic value of those carried out with an unblinded prototype showed weak outcome.

Hocagil et al. discussed this factor to be the main reason for weak sensitivity and specificity in their study and already reported about indications for an improper data processing within the device [13]. The results of the present study underline and support this assumption.

Linear to Hocagil et al., all raw data of false-positive or false-negative results were sent to the developer for retrospective analysis.

Since PneumoSonic Inc. assumed a software or antenna problem, an exchange unit with improved antenna- and processing-algorithms and with updated software was implemented after 22 patients. The revised prototype, however, did not lead to a significant increase of diagnostic accuracy (Table 6). After another 10 readings, a second exchange was carried out, with the same poor results. PneumoSonic Inc. finally suspected other reasons like external conditions to influence the readings.

However, external conditions like mechanical ventilation, obesity, or pulmonary diseases did not affect the results of PneumoScan (Table 5). Only mediastinal shifting as a consequence of a tension PTX significantly influenced PneumoScan-readings, a finding that could be crucial.

The basic usability of PneumoScan was satisfying, readings were quickly processed. Beside unreliable results, PneumoScan did not calculate PTX-volume or a more specific positioning within the thorax (e.g., apical, ventral, or lateral). The knowledge of PTX expansion and a more accurate positioning could be supportive in the decision process whether chest tube placement is necessary or not. Furthermore, the number of “preventive” chest tube placements, which might cause unnecessary delay and complications, could be reduced [14, 15]. In our case series PneumoScan detected one PTX with a maximal expansion of 0,5 cm, which needed no treatment. Another PTX (missed by PneumoScan) with the same expansion of 0,5 cm was treated with chest tube placement. Until now, it does not seem feasible to state a cut-off, where PneumoScan starts to detect a PTX. Even if the developer presents the PneumoScan with the phrase “…provides timely, objective results to a minimally trained operator” [16], the examiner still needs to assess the patients' medical condition for proper treatment.

Since especially ventral PTXs are difficult to detect, they are often missed in supine CXR [17]. Ding et al. found a weak sensitivity in their meta-analysis and so did our survey [3]. Despite low sensitivity of supine CXR to diagnose PTX, it remains an important tool in trauma management to quickly rule out rib or spinal fractures, pleural effusions, tracheal deviation, or mediastinal shifting and describe tube position.

The additional thoracic ultrasound was implemented in order to assess the feasibility of a competitive diagnostic bedside tool to PneumoScan. Today, ultrasound is getting more and more established in preclinical emergency medicine [18].

Overall diagnostic value of ultrasound to detect PTX is good, but different studies described examiner dependence, due to different levels of experience [3].

In our study, thoracic ultrasound was performed by two physicians, of which only one was trained according to DEGUM guidelines. The fluctuant findings in our study could therefore also rely on examiner skills. Furthermore, only lung-sliding and comet-trail-artefacts could have been evaluated with the B-mode ultrasound. Since the used ultrasound machine did not support M-mode, additional important signs such as “seashore sign” and “lung-point-sign” could not have been analyzed.

This study confirms that all examined methods to diagnose PTX have their limitations. It should be pointed out that clinical examination outperformed all other modalities. No device or examination method was able to detect PTX with an acceptable sensitivity. Only if all findings are considered together, a safe diagnosis can be made. Nevertheless, the present study has its limitations. Exclusion criteria often prevented the scanning of a patient with tension PTX, since all prehospital treated PTX were excluded. The patient's condition was described only by shock-index and peripheral oxygenation; however the need for catecholamines or fluids was not considered. Thereby, the patient's condition could have been falsified. Finally, the data of excluded patients were not saved due to the ethical vote and a statement concerning the condition of these patients was not possible. Thus selection bias could have occurred in this study.

## 5. Conclusion

The first survey with an unblinded prototype of PneumoScan implemented in the standard shock-trauma-room algorithm showed weak sensitivity and specificity in contrast to previous studies with a blinded device. To get a better understanding why PneumoScan showed such inconsistent results, further studies are needed. To rule out the issue of false PneumoScan readings, an analysis of the raw data of each reading with comparison to the displayed results would be essential. Furthermore, it would be desirable to include the intrathoracic PTX-expansion with volume calculation to see if certain PTX locations are more frequently missed and to verify the cut-off at which PneumoScan starts detecting a PTX. Since this study excluded all PTX patients who were treated by prehospital services, a prehospital-study could include patients on scene with significant larger PTX expansions, which might be easier to detect.

Until now we cannot recommend the general usage of PneumoScan. Nevertheless, MIR is a forward-looking alternative technology, with a high potential especially for disaster medicine and preclinical diagnosis.

## Figures and Tables

**Table 1:** Sensitivity and specificity of PneumoScan in previous studies.

	Levy et al. (trauma)	Levy et al. (surgical)	Albers et al.	van der Wilden et al.	Lindner et al.	Hocagil et al.
Patients	53	5	50	75	24	115
Sensitivity [%]	93.0	93.0	85.7	100	75.0	83.3
Specificity [%]	89.0	84.0	97.7	91.0	100	35.0

**Table 2:** Inclusion and exclusion criteria.

Inclusion	Exclusion
Age ≥ 18 years	Disruption of primary survey through study procedure
Suspected thoracic trauma	Life-threatening condition
Intended spiral CT scan	Preclinical PTX intervention

**Table 3:** * Patient characteristics*. All parameters are presented in relative and absolute values, or as median with 25th/75th percentile. The significant *p* value was calculated with Mann–Whitney *U* Test. ^a^Chi-Squared-Test. BMI: Body Mass Index; ISS: Injury Severity Score; AIS: Abbreviated Injury Scale; MAP: midarterial pressure; SpO_2_: peripheral oxygen saturation.

Parameter	Total	Non-PTX	PTX	*p*
Number	80	86,25% (69)	13,25% (11)	
Sex [m/f]	70%/30% (56/24)	71%/29% (49/20)	63,6%/3,4% (7/4)	.620^a^
Age [years]	49,0 (31,0/72,7)	50,0 (34,0/61,5)	31,0 (24,0/41,0)	**.005**
BMI [kg/m^2^]	25,9 (23,6/38,9)	26,2 (24,0/29,1)	23,2 (23,0/28,0)	.227
ISS	12,0 (9,0/22,0)	12,0 (9,0/17,5)	29,0 (20,0/41,0)	**.001**
AIS-Thorax	2,0 (2,0/2,8)	2,0 (1,0/2,0)	4,0 (3,0/4,0)	**.000**
Prehospital Intubation (*n*)	13,8% (11)	14,5% (10)	9% (1)	.629^a^
Intubation in time course (*n*)	6,3% (5)	4,3% (3)	18,2% (2)	.078^a^
Heartrate [bpm]	91,0 (80,0/104,0)	90,0 (79,5/100,0)	104,0 (97,0/114,0)	.012
MAP [mmHg]	99,0 (91,5/111,3)	101,6 (93.0/112,5)	93,3 (84,0/98,7)	.041
SpO_2_[%]	99,0 (96,0/100,0)	99,0 (97.0/100,0)	100,0 (96.0/100,0)	.947

**Table 4:** Diagnostic accuracy of all investigated methods (all data in percent); PPV = positive predictive value; NPV = negative predictive value.

	Sensitivity	Specificity	PPV	NPV	*n* =
Clinical examination	54,5	92,8	54,5	92,8	80
Chest radiography	27,3	100	100	89,6	80
Thoracic ultrasound	25	100	100	91,7	74
PneumoScan	20	80	13,3	86,7	75

**Table 5:** * Potential biases of PneumoScan-readings*. All parameters are presented in relative and absolute values, or as median with 25th/75th percentile. BMI: Body Mass Index; ISS: Injury Severity Score; AIS: Abbreviated Injury Scale. The significant value *p* was calculated with Mann–Whitney *U* Test. ^a^Chi-Squared-Test.

Parameter	Correct reading (*n* = 54)	False reading (*n* = 26)	*p*
Sex m/f [%]	70.4%/29.6% (38/16)	69.2%/30.8% (18/8)	.917 ^a^
BMI [kg/m^2^]	25.8 (23.6/28.8)	26.4 (23.2/30.4)	.700
ISS	12.0 (9.0/22.0)	17.0 (12.0/30.3)	.030
AIS-Thorax	2.0 (1.0/2.0)	2.0 (2.0/3.0)	.016
Prehospital intubation	14.8% (8)	11.5% (3)	.690^a^
Haematothorax	1.8% (1)	11.5% (3)	.076^a^
Contusion	13% (7)	15.4% (4)	.646^a^
Emphysema	1.8% (1)	7.7% (2)	.298^a^
Metastasis	0	3.8% (1)	.147^a^
Pleural effusion	1.8% (1)	3.8% (1)	.277^a^
Mediastinal shift	0	11.5% (3)	.039^a^
Rib fracture	18.5% (10)	38.5% (10)	.155^a^
Pericardial effusion	1.8% (1)	3.8% (1)	.593^a^

**Table 6:** Diagnostic accuracy of all used PneumoScan-devices (all data in percent); PPV = positive predictive value; NPV = negative predictive value.

Number	Readings	Sensitivity	Specificity	PPV	NPV	“No data”
1	22	20.0	80.0	25.0	75.0	2
2	10	-	80.0	-	100	-
3	48	20.0	80.0	11.0	89.0	3
